# Manure Refinement Affects Apple Rhizosphere Bacterial Community Structure: A Study in Sandy Soil

**DOI:** 10.1371/journal.pone.0076937

**Published:** 2013-10-14

**Authors:** Qiang Zhang, Jian Sun, Songzhong Liu, Qinping Wei

**Affiliations:** Institute of Forestry and Pomology, Beijing Academy of Agriculture & Forestry Sciences, Beijing, China; Catalan Institute for Water Research (ICRA), Spain

## Abstract

We used DNA-based pyrosequencing to characterize the bacterial community structure of the sandy soil of an apple orchard with different manure ratios. Five manure percentages (5%, 10%, 15%, 20% and 25%) were examined. More than 10,000 valid reads were obtained for each replicate. The communities were composed of five dominant groups (Proteobacteria, Actinobacteria, Chloroflexi, Acidobacteria and Bacteroidetes), of which Proteobacteria content gradually decreased from 41.38% to 37.29% as manure ratio increased from 0% to 25%, respectively. Redundancy analysis showed that 37 classes were highly correlated with manure ratio, 18 of which were positively correlated. Clustering revealed that the rhizosphere samples were grouped into three components: low manure (control, 5%) treatment, medium manure (10%, 15%) treatment and high manure (20%, 25%) treatment. Venn analysis of species types of these three groups revealed that the bacteria community difference was primarily reflected by quantity ratio rather than species variety. Although greater manure content led to higher soil organic matter content, the medium manure improved soil showed the highest urease activity and saccharase activity, while 5% to 20% manure ratio improvement also resulted in higher bacteria diversity than control and 25% manure ratio treatment. Our experimental results suggest that the use of a proper manure ratio results in significantly higher soil enzyme activity and different bacteria community patterns, whereas the use of excessive manure amounts has negative effect on soil quality.

## Introduction

China is the largest apple producer in the world,of which apple orchard cultivation area and yield were 2.06×10^6^ ha and 3.33×10^7^ tons, accounting for 43.78% and 47.86% of the world's production, respectively (FAOstat, 2010). However, 74% of China's total apple orchard area is distributed in hills, mountains and dry highland region, where limited soil organic matter (SOM) sustains crop productivity [1]. SOM can facilitate water entry [2], resist surface structure degradation and adsorb minerals and nutrients, all of which significantly impact fruit yield and quality. Manure improvement has been used in farmlands in China for thousands of years, and it is now the most effective manual way to increase SOM within apple orchards. Meanwhile, microbes within orchard soil is an important component of soil function [3] that are related with soil mineral nutrient release and plant disease resistance, and bacteria mineralization [4] is an important natural source of SOM. The evaluation of effects of manure application on the structure and functionality of soil microbial community is crucial to gaining a more holistic understanding of soil status under manure refinement.

Inspecting rhizosphere soil microbial community structure shifts after manure application is an important method for determining the proper way to improve ecosystem services and soil function. Bacteria are the most abundant and diverse group of soil organisms [5], and it is estimated that 1 g of soil contains more than 1,000,000 bacteria from thousands of different species. Since no more than 1% of these microbes can be cultured in the laboratory [6], the culture-based method cannot be used. The recent development of the microbial ecology base makes this investigation possible using partial ribosomal amplification and pyrosequencing techniques [7] to examine microbial diversity depth in virtually any sample type and detect changes within soil microbial groups. Soil profiles can then be compared using multivariate statistical techniques to reveal differences among microbial communities. Studies have shown that soil microbial communities were sensitive to management [8], seasonal changes [9], rhizosphere effects [10], pollution and the addition of composts and pesticides [11]. Qiu et al. [12] reported that bio-organic fertilizer changed the soil microbe community and reduced *Fusarium oxysporum* counts in cucumber plants. Sugiyama et al. studied [13] the soil bacteria communities of organic and conventional potato farms. Manter et al. [14] reported a highly diverse and cultivar-specific bacterial endophyte community in potato roots. Stéphane characterized rhizosphere soil bacteria profiles of oak [15] and found some rare phylogenetic groups. Lin et al. [16] found that different fertilizers had various impacts on soil bacteria. However, little was discovered about the apple rhizosphere microbial profile, and although manure treatment was employed in an earlier study, little was learned about the impact of different doses.

Our objectives were to determine the effects of manure improvement on apple rhizosphere soil and evaluate whether microbial community composition changes will directly affect microbial functionality as indicated by enzyme activities over a three-year study period. We hypothesized that soil bacteria community structures and bioactivity are affected by manure ratio.

## Materials and Methods

### Ethics statement

The experiment was carried out in our scientific research field for pomology studies which is owned by our institute, therefore, no specific permissions were required for these locations/activities, the field studies did not involve endangered or protected species.

### Soil sampling sites and collection

In spring 2009, sandy soil was purchased from a local construction materials market and sieved through a 5-mm screen. Different ratios of manure (0%, 5%, 10%, 15%, 20%, 25%) were then premixed into the sandy soil prior to nursery stock transplantation. One-year-old “Fuji” apple (“Red Delicious” × “Ralls Genet”) nursery with SH40 (*Malus honanensis*) as a dwarfing interstock and *Malus hupehensis* Var. Pingyiensis Jiang as a base stock was planted into 64 liter cubic Plexiglas boxes in triplicate. Soil samples were collected from depths of 0–40 cm in three different locations at 20-cm distances from the center of a 64 liter box using a 5-cm-diameter soil auger and transferred on ice to the laboratory at the beginning of autumn 2012. The soil samples were sieved through a 2-mm screen and homogenized prior to the analysis. One portion of the composite soil was stored in polythene bags and kept with dry ice for the molecular analysis, while another portion was used for the total N and SOM content and enzyme activity measurements.

### Soil enzyme activity characterization

Soil urease activity was detected using improved sodium phenate and sodium hypochlorite colorimetry. A total of 5 g of soil and 1 mL of methylbenzene were added to a 50-mL triangular flask and mixed. Fifteen minutes later, 10 mL of a 10% urea solution and 20 mL of citrate buffer (pH 6.7) were added to the mixture, the flask was shaken and the mixture was incubated at 37°C for 24 h. The solution was then filtered, 1 mL of the liquid was transferred to a 50-mL volumetric flask, 4 mL of 1.35 M sodium phenate and 3 mL sodium hypochlorite solution (0.9% active chlorine) were added and the flask was shaken slightly at constant volume. After 20 minutes, the solution was read at A_578 nm_ and the urease activity was calculated according to a working curve based on the nitrogen concentration of ammonium sulfate. The control solution contained no substrate. During the experiment, a control with no soil was also processed to rule out any system errors.

Saccharase and cellulase activities were estimated using the 3,5-dinitrosalicylic acid method according to previous report [17], all enzyme activity experiment was placed in triplicate.

### DNA extraction

Soil genomic DNA for the polymerase chain reaction (PCR) amplification was extracted from 0.5 g of soil using an E.Z.N.A Soil DNA Kit (Omega Biotek Inc., Norcross, GA, USA) following the manufacturer's instructions. DNA purity and concentration were analyzed spectrophotometrically using the e-Spect ES-2 (Malcom, Tokyo, Japan). The extracted DNA was stored at −20°C for up to 10 days before use.

### PCR amplification, quantitation, pyrosequencing

Bacterial 16 s rDNA was amplified by PCR to construct a community library using tag pyrosequencing. The bar-coded broadly conserved primers 27F and 533R containing the A and B sequencing adaptors (454 Life Sciences, Branford, CT, USA) were used to amplify this region. The forward primer (B-27F) was 5′-CCTATCCCCTGTGTGCCTTGGCAGTCTCAGAGAGTTTGATCCTGGCTCAG-3′, in which the B adaptor is underlined. The reverse primer (A-533R) was 5′-CCATCTCATCCCTGCGTGTCTCCGACTCAGNNNNNNNNNNTTACCGCGGCTGCTGGCAC-3′, in which the sequence of A adaptor is underlined and the string of Ns represents an eight-base sample-specific barcode sequence. Including the barcode and 454 primers, the length of the amplicon was approximately 596 nt. The PCR procedures were carried out in triplicate 20-μL reactions using 0.4 mM of each primer, 5 ng of template DNA, 1× PCR reaction buffer and 1.5 U of *TransStart FastPfu* DNA Polymerase (TransGen Biotech, Beijing, China). The amplification program consisted of the following: initial denaturation, 95°C for 2 min; 25 cycles of 95°C for 30 s (denaturation), 55°C for 30 s (annealing) and 72°C for 30 s (extension); and a final extension of 72°C for 5 min; and 10°C forever. PCR products of the same sample were assembled and visualized on 2% agarose gels (TBE buffer) using ethidium bromide and further purified using a gel extraction kit (TransGen). The DNA concentration of each PCR product was determined using a Quant-iT PicoGreen double-stranded DNA assay (Invitrogen, Darmstadt, Germany) and was quality controlled on an Agilent 2100 Bioanalyzer (Agilent Technologies, Palo Alto, CA, USA). The PCR products from each reaction mixture were pooled in equimolar ratios and then subjected to emulsion PCR to generate amplicon libraries. Amplicon pyrosequencing was performed from the A-end using a 454/Roche A sequencing primer kit on a Roche Genome Sequencer GS FLX Titanium platform at Majorbio Bio-Pharm Technology Co., Ltd. (Shanghai, China). Complete data sets are submitted to the NCBI Short Read Archive under accession no. SRX337490.

### Statistical and bioinformatics analysis

Rarefaction analysis and Good's coverage for the libraries were determined using custom scripts on R version 2.15.2 (R Foundation for Statistical Computing). Heatmap figures were generated using Gene cluster and Tree View (written by Michael Eisen, Stanford University). Venn diagrams curves were created with the online tool Venny (Juan Carlos Oliveros; http://bioinfogp.cnb.csic.es/tools/venny/index.html) and Canoco 4.5 was used to run a redundancy analysis (RDA). Analysis of variance was performed using SPSS Statistics 18 (IBM, Armonk, NY, USA). Community richness index, community diversity index, data preprocessing, operational taxonomic unit-based analysis and hypothesis tests were performed using mothur (http://www.mothur.org/). The histogram was created using Microsoft Excel 2007 (Microsoft, Redmond, WA, USA).

## Results and Discussion

### Soil properties and enzyme activity

Three years after planting, soil was collected and physicochemical properties were tested (Table 1). The SOM contents of the apple rhizosphere soil correlated with manure ratio except that the 20% manure treatment was 17.30 g/kg, which is higher than that of the 25% manure treatment. Available contents of the soil nutrients P, K, Fe, Zn and B content were increased by manure application, while that of Ca was not. Ca is known to be an important factor affecting apple root and shoot growth [18], and all manure treatments decreased available Ca content compared to control and increased plant mass growth (data not shown), probably because manure application enhanced plant growth and increased Ca consumption. Cation exchange capacity (CEC), the number of positive charges that a soil can contain, was mainly influenced by SOM and pH [19]. In our experiment, although SOM content increased stepwise with manure ratio and the pH of all treatment soils was 8.43–8.64, the 15% manure treatment had the highest CEC, indicating that overdose levels of manure might have a negative impact on soil fertility. This is in accordance with a former study which showed higher ratio ash admixture to compost changed apple rhizosphere microbe communities but did not enhance C utilization as lower dose [20].

**Table 1:** Soil properties under different manure refinement levels.

Manure Ratio	Total N	Organic Matter	Available N	Available P	Available K	Available Ca	Available Fe	Available Zn	Available B	Cation Exchange Capacity	pH
(%)	(g/kg)	(g/kg)	(mg/kg)	(mg/kg)	(mg/kg)	(g/kg)	(mg/kg)	(mg/kg)	(mg/kg)	(mmol/kg)	
0	0.994±0.009^c^	2.09±0.04^a^	29.8±0.3^a^	30.8±2.0^a^	78±3^a^	2.77±0.06^d^	4.98±0.10^a^	4.77±0.06^b^	0.21±0.02^a^	160±7^a^	8.63±0.01^c^
5	0.876±0.004^b^	4.38±0.04^b^	64.3±0.8^e^	67.7±2.5^b^	120±5^b^	2.61±0.02^c^	9.59±0.12^b^	3.97±0.02^a^	0.25±0.02^a^	151±6^a^	8.64±0.02^c^
10	1.97±0.11^d^	6.63±0.13^c^	35.7±1.1^b^	85.5±2.8^c^	200±9^c^	2.36±0.09^b^	10.3±0.2^c^	5.32±0.11^d^	0.49±0.04^b^	154±7^a^	8.43±0.02^a^
15	0.718±0.011^a^	8.21±0.04^d^	46.4±0.9^d^	150.0±2.0^d^	342±8^d^	2.16±0.04^a^	13.0±0.2^d^	5.15±0.06^c^	0.66±0.03^c^	181±6^b^	8.52±0.08^b^
20	0.975±0.010^c^	17.30±0.44^f^	40.5±1.4^c^	161.1±10.6^e^	334±5^d^	2.33±0.03^b^	14.3±0.5^e^	8.95±0.09^f^	0.63±0.02^c^	144±6^a^	8.50±0.02^b^
25	0.775±0.016^a^	12.20±0.26^e^	47.6±0.9^d^	196.2±4.3^f^	420±6^e^	2.35±0.04^b^	16.1±0.8^f^	7.31±0.03^e^	0.86±0.02^d^	152±10^a^	8.59±0.03^c^

Averages of replicates ± standard error; means followed by different letters are significantly different at *P<*0.05.

**Figure 1:**
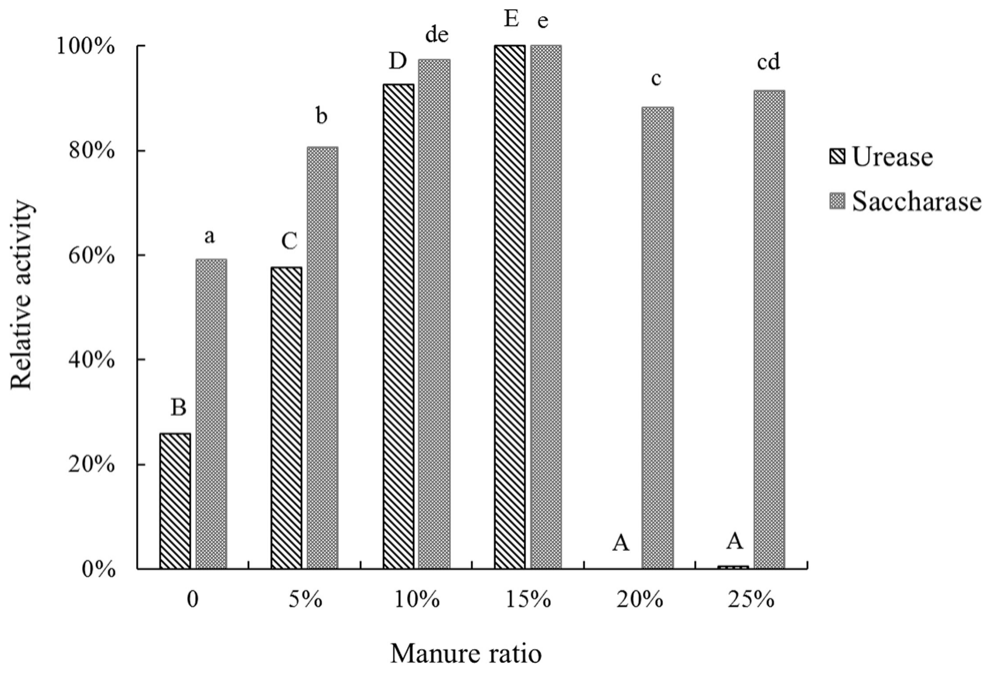
Urease and saccharase activities of soil with different manure ratios. Each enzyme's highest activity levels was defined as 100%. Different letters means significantly different at P<0.05, urease is represented as capital letters.

Hydrolytic enzymes regulate the rate at which organic materials are degraded and nutrients become available to plants, thus reflecting soil fertility [21]. Soil urease, saccharase and cellulase activity were tested (Fig. 1). Urease activity was increased by manure application, but once the manure ratio was ≥20%, urease activity was heavily inhibited. In an earlier study, urease content was reportedly increased by the use of both organic and inorganic fertilizers [22], [23], while it was significantly correlated with SOM and total N content. However, we found that the use of overdose manure levels significantly inhibited urease activity. Saccharase has been shown to be an important factor affecting soil alkali-hydrolyzable N [24], while our experimental results showed a similar trend to that of available N in that, 15% manure treatment resulted in the highest value (relatively high soil fertility). High manure doses might change the soil microbe community structure and reduce soil enzyme activity. Cellulase was not detected in any of our treatments.

### Pyrosequencing and sequence analysis

A total of 162,141 valid reads and 67,714 operational taxonomic units (OTUs) were obtained from the 18 samples through 454 pyrosequencing analysis (Table 2). After excluding non-ribsomal DNA, low quality (containing fuzzy bases) or short sequences (reads <200 bp long), mothur was used to assign OTUs at the 97% sequence similarity level and to calculate Shannon and Simpson's diversity indexes, Chao, Ace and Good's coverage. Total reads and OTUs did not differ significantly among treatments, so the sequencing depth was unique for each sample. The bacterial diversity richness indexes ACE and Chao at the 3% dissimilarity level tended to be higher under manure treatment than the control; however, it is important to emphasize that 95% confidence interval calculations for the two indexes showed that none of these trends were significant. On the other hand, the Simpson indexes for 5%, 10%, 15% and 20% manure treatment were lower than those for the 25% manure treatment and the control, indicating that these treatments led to higher bacterial diversity. Good's coverage was around 0.75 for all samples, indicating a 75% species detection rate which was normal for pyrosequencing of soil microbes [25].

**Table 2:** Comparison of the estimated operational taxonomic unit (OTU) richness and diversity indexes of the 16S rRNA gene libraries for clustering at 97% identity as obtained from the pyrosequencing analysis.

Manure ratio	Reads	OTU	ACE	Chao	Coverage	Shannon	Simpson
0%	9015±2997^a^	3533±812^a^	9581±2336^a^	6816±1559^a^	0.767±0.024^a^	7.58±0.112^a^	0.0010±5.7735^b^
5%	9485±2305^a^	4032±878^a^	1101±2941^a^	7754±1732^a^	0.749±0.005^a^	7.776±0.179^a^	0.0007±5.7735^a^
10%	8501±1153^a^	3643±478^a^	9814±1751^a^	7204±1217^a^	0.747±0.026^a^	7.706±0.117^a^	0.0008±5.7735^a^
15%	9138±1096^a^	3871±213^a^	1069±835^a^	7648±393^a^	0.749±0.019^a^	7.766±0.005^a^	0.0007±0.0001^a^
20%	9916±1429^a^	4136±496^a^	1105±1744^a^	8133±1134^a^	0.755±0.032^a^	7.836±0.116^a^	0.0007±0.0001^a^
25%	7991±768^a^	3354±393^a^	9651±2464^a^	6903±1290^a^	0.749±0.019^a^	7.573±0.081^a^	0.0010±5.7735^b^

Averages of replicates ± standard error; means followed by different letters are significantly different at *P<*0.05.

Nacke et al. [26] reported that for forest soil, with >20,000 reads per sample, the rarefaction curve was not saturated until genetic distances of 25% rather than 3% were used. In contrast Kunin et al. [27] suggested that a 97% clustering threshold is necessary for reliable estimation of community diversity. However, the pyrosequencing data gave much more information than the traditional microbe culture method or the molecular cloning library, and it effectively showed differences between treatments and revealed that manure application changed the soil microbial diversity in this study.

### Taxonomic composition analysis

**Figure 2:**
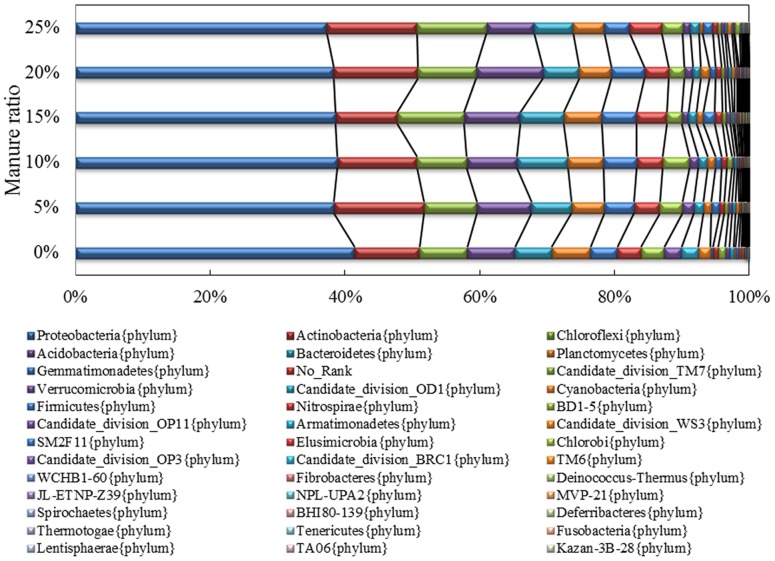
Comparison of the bacterial communities at the phylum level. Relative read abundance of different bacterial phyla within the different communities. Sequences that could not be classified into any known group were labeled “No_Rank”.

All of the sequences were classified to 39 phyla or groups by the mothur program. The predominant bacterial components of the different treatments were similar, while the distribution among phyla and groups varied (Fig. 2). Proteobacteria, Actinobacteria, Chloroflexi, Acidobacteria and Bacteroidetes were the five most dominant phyla in all treatments, accounting for >70% of the total reads. Proteobacteria presented the highest percentage in all treatments, a finding that supports those of other reports [25], [28]. Most Proteobacteria bacteria are Gram-negative and many are responsible for legume symbionts nitrogen fixation [29] and polycyclic aromatic hydrocarbons [30], Proteobacteria bacteria are widely distributed in soil samples including contaminated soil [31].

A wide variety of pathogens are also Proteobacteria, such as Agrobacterium, Escherichia, Salmonella and Helicobacter. In the present study, Proteobacteria content gradually decreased from 41.38% to 37.29% as manure ratio increased from 0% to 25%, respectively. Relatively low Proteobacteria distribution (23%) was observed in an area of environmentally protected soil through metatranscriptome [32], while an earlier study of a cucumber field [12] suggested that the use of manure composts with antagonistic microorganisms decreased Proteobacteria distribution from 40% to 20%, suppressed disease incidence by 83% and reduced yield losses. Hence, we presumed that using manure treatment to reduce the Proteobacteria ratio was beneficial for apple plants.

Actinobacteria are Gram-positive bacteria that are widely distributed throughout the soil and water ecosystem. In many studies, Actinobacteria ratios are less than Proteobacteria ratios in soil samples [12], [33]; however, Actinobacteria was the most abundant phylum in sludge [34] and dry land [35], probably because the secondary metabolites produced by Actinobacteria enhanced its adaptability. The lowest Actinobacteria content (9.09%) was detected after 15% manure treatment, so we assume that 15% manure application supplied a better nutrition environment for most of the bacteria.

The proportion of Chloroflexi was not more than 5% of the soil bacteria community in many pyrosequencing studies [33], whereas it increased under heat-exposed conditions [36]. In the present study, it was approximately 10% in all treatments, probably because Chloroflexi includes many aerobic thermophile members while the sandy soil led to better oxygen supply.

Acidobacteria is another abundant bacterial phylum that was recently discovered within soils. The majority had not been cultured but studies have shown that the phylum was negatively correlated with environmental pH [37]. In the present study, the Acidobacteria ratio did not differ significantly among the different treatments, probably since the soil pH values was similar.

**Figure 3:**
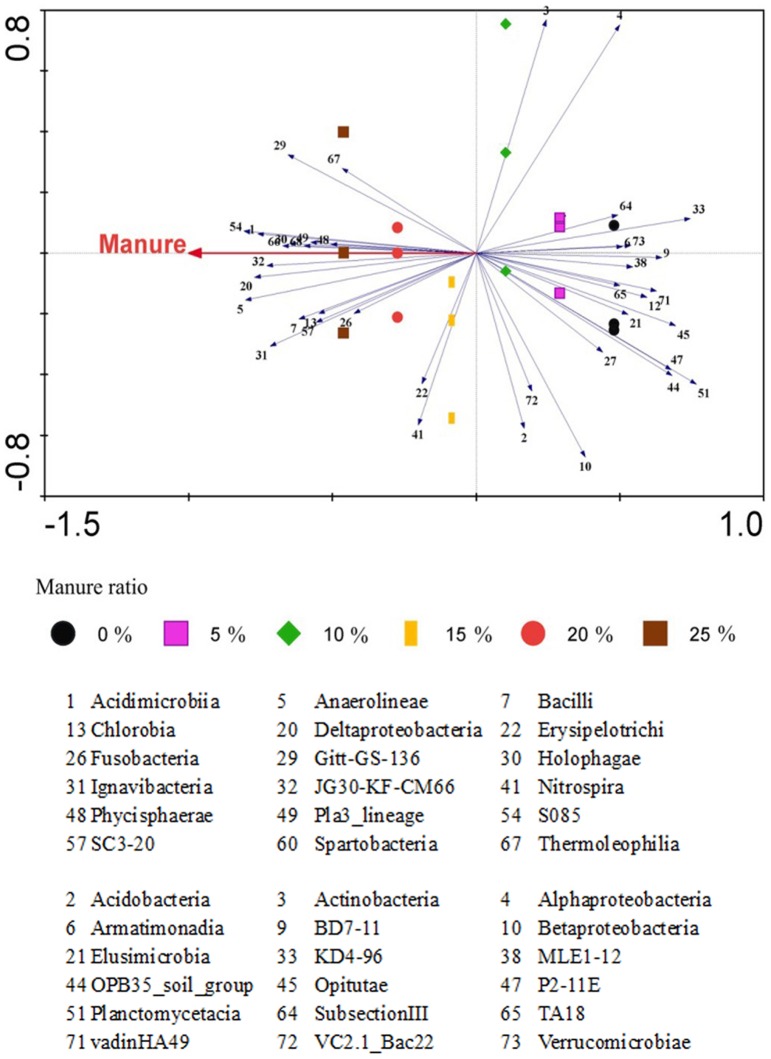
Redundancy analysis on the relative abundance of bacterial class using Canoco 4.5. The canonical axis (horizontal axis) shows that with 21.5% total variability, the correlation with manure ratio is 0.909. The 37 classes that passed the criterion of having >21.5% variability are shown on the horizontal axis.

Due to the relative abundance of bacterial classes, RDA was performed to check the effect of manure ratio using Canoco 4.5 (Fig. 3). The canonical axis (horizontal axis) explains 21.5% of the total variability and a correlation with manure ratio of 0.909; thus, the horizontal axis highly represents the influence of manure ratio. A total of 37 classes passed the criterion of having >21.5% variability in its values explained by the horizontal axis. Of these, Acidimicrobiia, Anaerolineae, Bacilli, Chlorobia, Deltaproteobacteria, Erysipelotrichi, Fusobacteria, Gitt-GS-136, Holophagae, Ignavibacteria, JG30-KF-CM66, Nitrospira, Phycisphaerae, Pla3_lineage, S085, SC3-20, Spartobacteria and Thermoleophilia were positively correlated with manure ratio, while Acidobacteria, Actinobacteria, Alphaproteobacteria, Armatimonadia, BD7-11, Betaproteobacteria, Elusimicrobia, KD4-96, MLE1-12, OPB35_soil_group, Opitutae, P2-11E, Planctomycetacia, SubsectionIII, TA18, vadinHA49, VC2.1_Bac22 and Verrucomicrobiae were negatively correlated.

**Figure 4:**
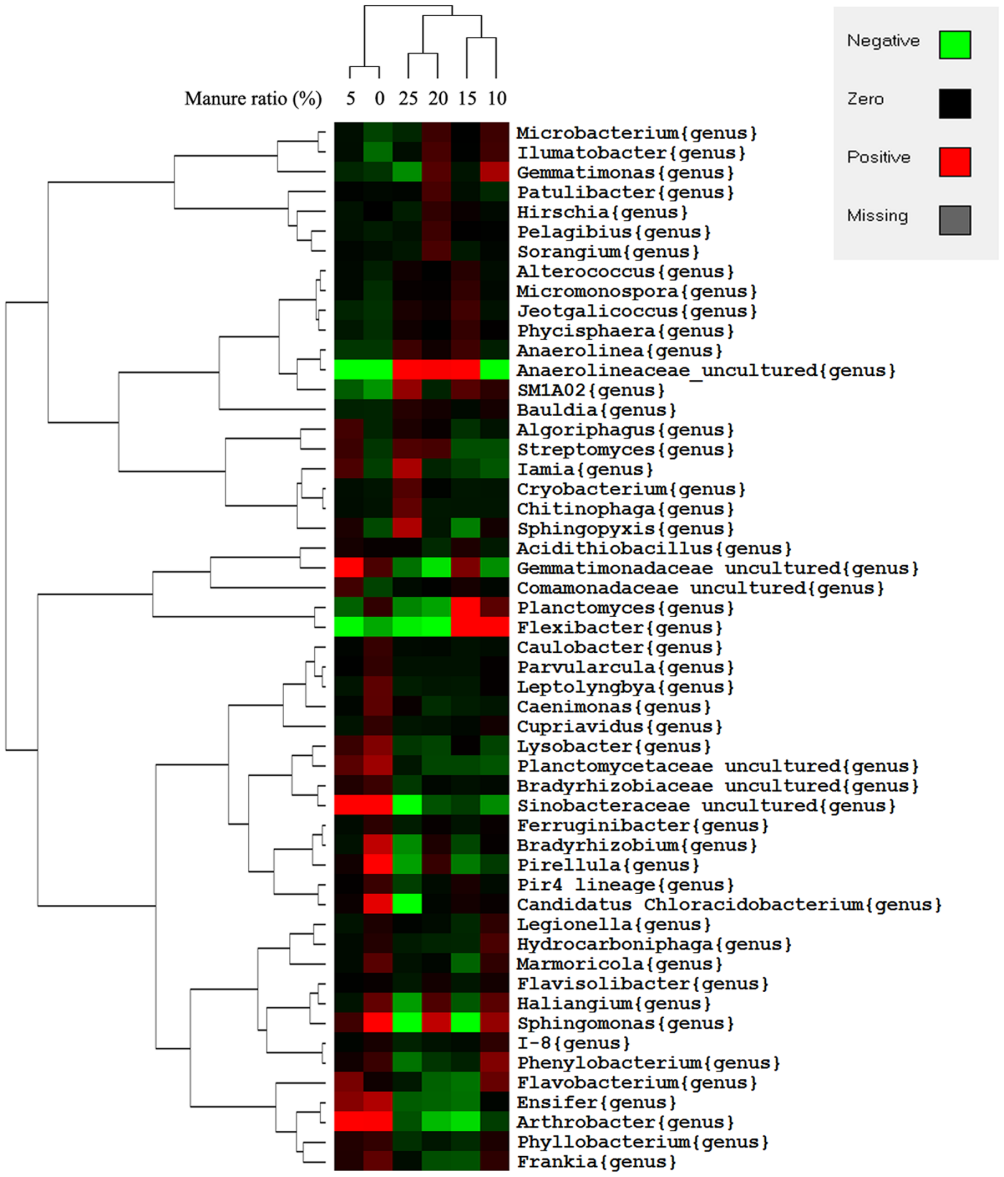
Genus level bacterial distribution among the six samples. The phylogenetic tree was calculated using the neighbor-joining method, while the relationship among samples was determined using Bray distance and the complete clustering method. The heatmap plot was generated by Gene cluster and Tree View (written by Michael Eisen, Stanford University). Genuses with >0.1% relative abundances are quoted and the species were mean centered before clustering.

Overall, 231 genera were found across the samples. All of the genera data underwent ANOVA by SPSS, and 82 genera showed significant difference among treatments, of which 53 genera with abundance >0.1% were used to cluster the samples and a heatmap was generated using Treeview (Fig. 4). The phylogenetic tree was calculated using the neighbor-joining method, while relationships among samples were determined by Bray distance and the complete clustering method. Samples were clustered into three groups, the low manure group (LM; 0% and 5% manure), medium manure group (MM; 10% and 15% manure) and high manure group (HM; 20% and 25% manure).

*Anaerolineaceae* was reported to be found in many samples including soil, sludge and water samples [38], and little was known about this genus except for its anaerobism, probably because the difficulty of culturing anaerobic bacteria. One earlier pyrosequencing revealed that it was more frequently encountered in anaerobic environments [39]. In this study, *Anaerolineaceae* increased with manure ratio from 1.56% (0% manure) to 3.83% (25% manure). We presume that this was due to the sandy soil and that the higher SOM led to lower oxygen supply, thus increasing the *Anaerolineaceae* ratio.

Ratios of *Sinobacteraceae*, *Ensifer* and *Arthrobacter* were higher in LM treatment. *Sinobacteraceae* consisted of Gram-negative, non-motile, rod-shaped bacteria, and a strain was isolated from polluted soil sample by enriched culture. The genus, which is represented in many contaminated soil samples [40], [41], decreased from 1.65% (0% manure) to 0.66% (25% manure). *Arthrobacter* is also commonly found in soil. All species in this genus are Gram-positive obligate aerobes that are rod-shaped during exponential growth and cocci in the stationary phase. This genus was also involved in the soil bioremediation of polluted environments. In an earlier study, Mazzola [42] reported that the proportion of *Arthrobacter* in apple rhizosphere decreased naturally as the plant grows. One explanation could be that plant growth benefited under manure application, which impacted microbial diversity.

*Ensifer*, also called *Sinorhizobium,* is a genus of N-fixing bacteria (rhizobia), and the decreasing *Ensifer* content reflective of high nutrient levels reduced the N fixing capacity of the soil microbes. *Flexibacter* is well known for its yellow-hued species that are identified as fish pathogens [43]; however, in soil, *Flexibacter* species such as *Flexibacter canadensis* have a denitrification [44] function in which they denitrify NO_3_^−^ and NO_2_^−^ to gaseous forms with increased oxygen tolerance. The higher content of *Flexibacter* in MM compared to LM and HM was in accordance with soil urease activity and indicative of a higher nitrous oxide reductase level. It has been reported that integrated apple farms maintained higher level of *Flexibacter* than organic farms [45] since denitrification causes nutrient loss, so the dynamic trend of *Flexibacter* indicates that the impact of manure application on apple rhizosphere soil was not linear. The profile differences of LM, MM and HM also fit the soil enzyme results (Fig. 1) in which MM showed highest urease and saccharase activities.

**Figure 5:**
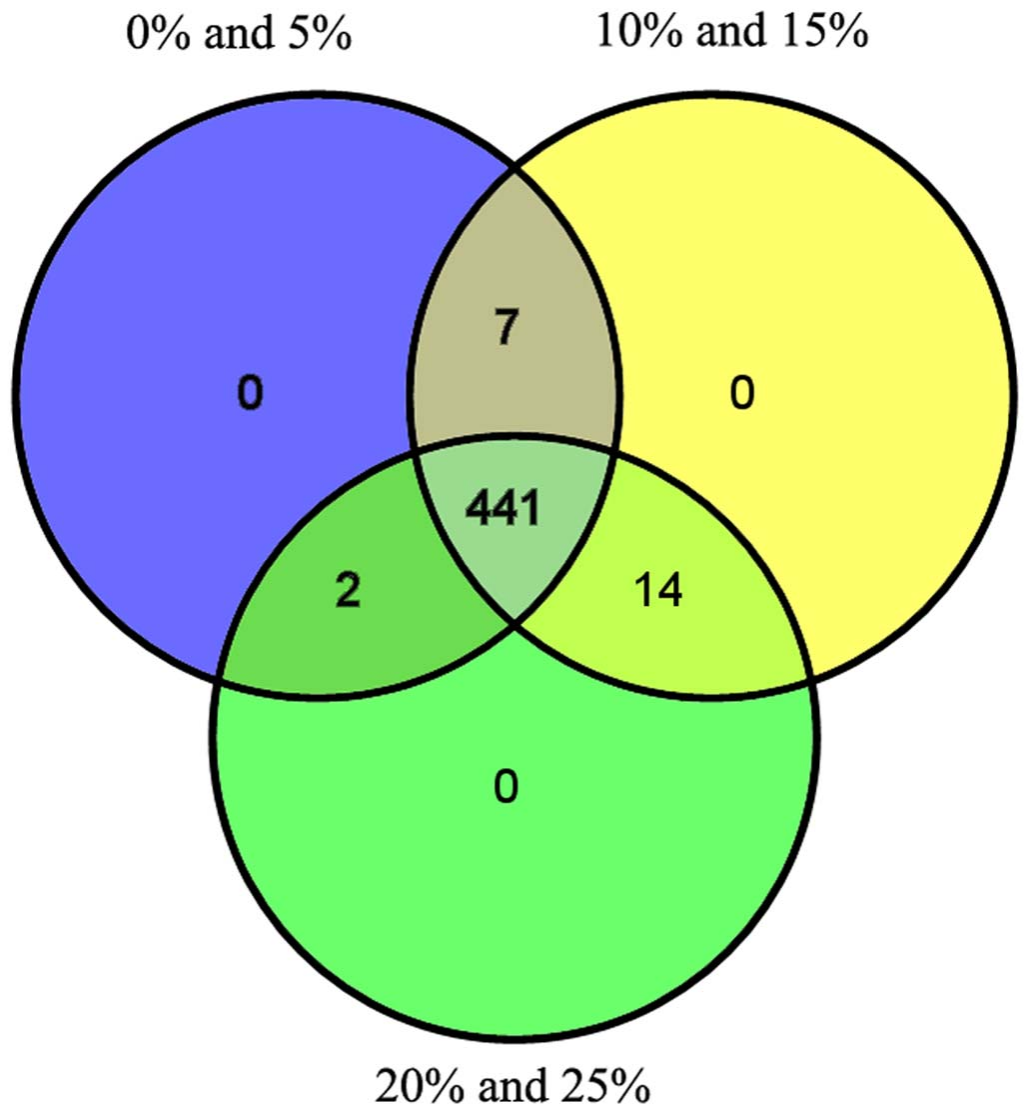
Shared species analysis of the different treatment groups. The Venn diagram shows the unique and shared species among the different treatments.

A Venn diagram based on species level comparing the LM, MM and HM groups revealed that most species were seen in each group; thus the bacterial community differences were mainly reflected by ratio rather than species variety (Fig. 5). However, most species showing different distributions among groups were uncultured. This finding indicated that pyrosequencing could be used to discover many uncultured bacterial species.

In the present study, we employed high throughput pyrosequencing to characterize apple rhizosphere soil microbial communities under different doses of manure applied after three-year culturing. The study results indicated that different manure ratios resulted in different changes in soil enzyme activity and bacterial communities, and that the use of overdose levels of manure maintained different bacteria community structures and decreased soil enzyme activity.

In conclusion, our study findings support the hypothesis that manure improvement exerts significant effect on soil enzyme activity and bacterial communities. Mid-level manure improvement increased soil enzyme activity and bacterial diversity, whereas over-dose levels of manure had an adverse impact.
